# Experiences of migrant parents of children with special health and welfare needs in Nordic countries: a scoping review

**DOI:** 10.1177/14034948241277057

**Published:** 2024-09-28

**Authors:** Archlove Takunda Tanyanyiwa, Victor Chimhutu, Henning Pettersen

**Affiliations:** 1 Inland Norway University of Applied Sciences, Institute of Social Work and Guidance, Lillehammer, Norway; 2 Inland Norway University of Applied Sciences, Department of Public Health and Sports Sciences, Elverum, Norway

**Keywords:** migrants, caring responsibility, disability, health and welfare, social inclusion

## Abstract

**Study aim:**

The objective of this scoping review was to explore and summarise emerging themes in existing studies on personal experiences of migrant parents of children with special health and welfare needs and their interaction with health and welfare services in the Nordic countries.

**Methods:**

A comprehensive and systematic search for relevant articles in electronic databases was conducted in PubMed, PsycINFO and Web of Science between 2 April 2023 and 31 May 2023. The scoping review followed identified guidelines in conducting scoping reviews. Out of the initial 1836 study results, 62 studies were read and assessed as full text and a total of 13 studies met the inclusion criteria.

**Results:**

Using thematic analysis, three key thematic categories were identified: (a) Initial responses to having a child with health and welfare challenges; (b) encountering the Nordic health and welfare services; (c) implications on social inclusion. The challenges to participation faced by migrants threatened their wellbeing, impacted their relationships and influenced their coping strategies and opportunities for social inclusion.

**Conclusions:**

**The Nordic countries have accessible, affordable and well-equipped infrastructure for health and welfare services compared with the home countries of most migrants. Future research is necessary to explore alternative strategies and spaces to promote participation and involvement of migrant parents of children with special health and welfare needs to inform public health and welfare services development and research**.

## Introduction

Providing care for children with special health and welfare needs can be more demanding and stressful for migrant parents and their families. By definition, children with special health and welfare needs encompass those who have or are at risk of chronic physical, developmental, behavioural or emotional conditions that require higher levels of care and dependency than ordinarily required by other children [1]. The United Nations (UN) defines a migrant as any person who has changed his or her country of residence regardless of their nature or motive of movement [2]. In this scoping review, we consider migrants to be people who come to a Nordic country (host country) from outside Western Europe to stay legally in their respective host countries, excluding those living in asylum-seeking institutions.

Over the past two decades, there has been a significant growth in the migrant population globally [3]. The Nordic countries, that is, Norway, Finland, Iceland, Denmark and Sweden, are no exception to this demographic growth [4–7]. As of 2020, the total population of the Nordic region had increased to 27.2 million, where 13.3% are migrants and 3.7% have parents with a migrant background [8]. Migrants from the Middle East/Asia are represented more highly in the Nordic region compared with other Western countries [9]. Notably, there are more migrants from Syria (260,000) in Sweden, Denmark and Norway than in any other country [8]. As of 2019, there has also been a rapid increase in Africans in the Nordic region, with about half of those coming from Somalia (120,000) and Eritrea (71,000) [8, 10]. Although there are multiple reasons for migration, such as work and education, the increase of migrants in the Nordic region is attributed largely to the refugee crises of the past two decades [9, 10].

This increase in the migrant population has resulted in health and welfare systems of these countries requiring adjustments in order to serve their diversified population [5, 7, 11–13]. Despite the efforts to adjust welfare policies and practice, studies have shown that migrants with non-Western backgrounds experience greater risk of poorer health outcomes compared with those from mostly Europe [13, 15–17]. Some migrants are parents of children with special health and welfare needs, referred to in this scoping review as Migrants with Caring Responsibilities (MCR). First, due to caring responsibility demands, the lives of MCR end up revolving around caring for the child’s special health and welfare needs, which may result in social isolation [18]. This usually leads to financial limitations, shame or stigma and diminishing opportunities for integration into society [17, 19]. Second, many MCR are faced with the stress that comes with building the knowledge and familiarity needed to navigate through unfamiliar health and welfare systems in their host countries [20, 21]. In addition, these experiences are linked to combinations of interrelated factors, including length of stay, reasons for migration, characteristics of country of origin and social status [5, 22, 23]. According to Kvarme et al. [18], such complex challenges might not only alienate MCR but also their children, which may result in social exclusion.

The Nordic countries are arguably a global benchmark because of, among other reasons, their largely state-funded health and welfare system [24]. Such welfare systems take universalistic and egalitarian approaches to public services [24, 25]. Their welfare approaches are credited for a high quality of life among their populations due to their accessibility and affordability [7, 18, 24, 26]. However, previous studies have reported that the Nordic health and welfare approach to service may be more prescriptive, which limits the agency of MCR who have to adjust to systems and models of care different from those of their countries of origin [27, 28]. Tøssebro argues that, although the Nordic countries have relatively similar welfare models, they have differences in policy and practice [23]. Regardless of the differences between the backgrounds of individual migrants and services between countries, migrants collectively remain a demographic group at greater risk of poorer health outcomes [11]. Given this background, conducting an exploration of studies that focus mainly on experiences of MCR as service users may contribute to future research and service development in health and welfare.

We did not find previous scoping reviews that assess the breadth of existing research on the experiences of MCR within the Nordic region. Therefore, these experiences need to be elevated both to identify existing gaps in research and inform health and welfare services development [29]. Exploring the breadth of existing studies that focus on experiences and perspectives of MCR as consumers of services could help in identifying the existing gaps in both research and practice [30].

The rationale behind this study is further strengthened by the views of scholars such as Whitmore and Snethen [31], who argue that more attention is needed and extra support expected from family, health and social services in monitoring and providing assistance to migrant children with special health and welfare challenges. This scoping review aims to explore and summarise emerging themes in the existing peer reviewed studies on the personal experiences of migrant parents of children with special health and welfare needs and their interaction with health and welfare services in the Nordic countries. The article traces some key empirical findings and developments within health and welfare by means of a scoping study of peer-reviewed, scholarly articles published between 2008 and 2023.

### Contextualising and defining healthcare and welfare services in the Nordic countries

We use Norwegian welfare legal standards, which refer to service users more broadly and include both medical access and persons who use the health and welfare system as non-patients [32]. We consider special health and welfare services to be public health services, e.g., clinical diagnosis and related biomedical support to both the parents and their children, including services that may not be classified as healthcare [32]. These services may involve institutions that provide social security and employment benefits, child protection services, schools and kindergarten or leisure services. Thus, these services encompass interventions meant to support not only the children, but also the MCR or families involved in coping with the caregiving role [33].

## Conceptual and theoretical background

We used *social inclusion* as a conceptual framework for this scoping review. Social inclusion is broadly defined as an attempt to *‘break down barriers that prevent full participation’* [34]. According to Ainscow [35], social inclusion in welfare would involve consistent efforts to respond to diversity by collecting and evaluating information in order to proffer improvements in policy and practice. This scoping review will incorporate three major forms of social inclusion, conceptualised by Gidley et al. [36] as key strategies to respond to diversity in a population and ensure the social inclusion of vulnerable groups in society. These strategies are outlined as *access*, *participation* and *empowerment* [36]. These three strategies are conceptualised in other studies as components, perspectives or forms of social inclusion and empowerment [37–42]. These different views of social inclusion influenced our interpretation of findings, discussion and conclusions.

### Social inclusion as access

Access entails the presentation or existence of opportunities that facilitate the ability of the MCR to harness resources such as social support, networks and information about available opportunities and services [42–44]. Bexley et al. describe these as community resources and link them to the social capital required to facilitate the social inclusion of vulnerable groups [44]. Access can extend further beyond the availability of opportunities for participation to include an investment of resources to limit barriers to the full maximisation of those opportunities available for inclusion [41]. We use access both as a perspective of social inclusion of MCR with health and welfare services and as a form of strategy for social inclusion.

### Social inclusion as participation

Social inclusion in health and welfare can also be explained within the context of participation and community engagement [43]. According to Eide et al. [39], participation is a continuous process of interaction between individuals, groups and the environment. They argue that participation may create opportunities for relationships building between MCR, society and the system that manages it [39]. Participation can be further described at different levels, that is, individual, group or societal [39]. Strategies for participation and engagement such as mentoring, learning networks, leisure activities, arts and sports interventions are also recommended [36, 45].

### Social inclusion as empowerment

Empowerment can be understood as an attempt to maximise the potential of individuals to take responsibility and control over their lives [36, 46]. Empowerment is an extended and advanced form of participation that involves the shift of power and responsibility into the hands of the vulnerable or disadvantaged groups [40]. Empowerment can also be understood, according to Arnstein [37], as a form of citizen power where partnership and delegation of power exist in society. Empowerment strategies, such as co-production and user-involvement in service development, are identified as ways to empower MCR [40]. Social inclusion is therefore an ongoing collaborative and empowering process [43].

Overall, social inclusion is based on social justice, facilitation and provision of empowering tools and resources that promote access to the social capital of the dominant culture [43]. In this review study, we will use the social inclusion perspective to discuss the experiences of MCR with health and welfare services and their integration into Nordic society.

## Methods

The review followed relevant ethical guidelines and consideration in conducting scoping reviews. In addition, this study is registered at the Norwegian Agency for Shared Services (SIKT) in Education and Research, reference no. 475905.

### Review design

A qualitative scoping review design was selected for this study. Scoping reviews are an explorative and broad overview of what exists pertaining to a specific topic, irrespective of study quality [29, 47]. According to Arksey and O’Malley [47], a systematic review typically focuses on a more specific question, whereas a scoping study address broader topics where many different study designs might be applicable. It was useful for us to use a scoping review approach to examine the study [29, 47]. The study will summarise findings from existing studies with the goal of identifying gaps in the literature to in aid the planning of future research, including a systematic review if necessary [29, 48]. A scoping review was chosen to shed light on the Nordic public health and welfare services for MCR and, more broadly, the social inclusion of migrants.

In order to achieve our goal, we used Arksey and O’Malley’s framework for scoping reviews [47], which involves identifying the research question, selecting relevant studies, charting the data, and summarising and reporting the results. The PRISMA (Preferred Reporting Items for Systematic reviews and Meta-Analyses) recommendations for scoping reviews further informed how the above-outlined framework was implemented [29].

### Search strategy

A preliminary basic search to identify a wide range of scoping reviews and articles beyond the Nordic countries was conducted. Studies that use either migrant or immigrant were considered in the preliminary search as both terms are used interchangeably in most of the studies reviewed during the preliminarily search stage. A comprehensive and systematic search for relevant articles in electronic databases was conducted in *PubMed*, *PsycINFO* and *Web of Science* between 2 April 2023 and 31 May 2023. The different terms and combination of phrases we used were as follows: PARENTS/MOTHERS/ FATHERS/FAMILIES; IMMIGRANT, MIGRANT, HEALTH, AND WELFARE SERVICES/SYSTEMS/PROGRAMS; CHILDREN/ADOLESCENT; DISABILITY/COM-PLEX HEALTH NEEDS/SPECIAL NEEDS/HEALTH CHALLENGES.

Previous studies have acknowledged the significance of existing knowledge resources and networks in generating relevant sources for scoping review study design [49]. As a result, the initial systematic search results and ongoing search were presented, and feedback was given through affiliated research groups and research communities. We also explored work by colleagues in the same research field. Other strategies used included a manual search of the reference lists of all relevant studies.

### Quality assessment

The quality of the whole scoping review was examined through a rigorous process that followed carefully identified guidelines in conducting systematic reviews [29, 47]. However, the review did not assess quality based on the depth of the individual research studies because the primary goal of a scoping review, as asserted by Arksey and O’Malley [47], is to seek breadth rather than depth.

### Study selection procedure

The initial selection stage involved inclusion by study title, that is, articles that had major key search words involving migrants, children with disabilities/special health needs and experiences with health and welfare services. Second, all articles that matched the inclusion criteria were assessed and considered. Using PRISMA guidelines, abstracts, the location of the study, methodology, and sample characteristics were considered in the assessment process. The primary author took responsibility for the initial process with co-authors accessing the eligibility of the selected studies. Uncertainties regarding what to exclude or include were deliberated with co-authors, and a clear justification for the inclusion and exclusion was set.

### Inclusion and exclusion criteria for the selected studies

The scoping review considered previous research with primarily qualitative or mixed methods research designs (Table I). Studies focusing on experiences of migrant parents of children with special health and welfare needs, including parent’s needs, expectations, participation and coping as MCR, were included. Primarily, qualitative and mixed studies were included over exclusively quantitative studies because they provided an opportunity to explore the subjective opinions, emotions and perceptions of experiences of MCRs, which might be underexplored through quantitative approaches. Studies that explore only service providers’ perspectives on MCR experience without the voices of migrants themselves were excluded from this study. All inclusion criteria had to be met. The literature search process, however, did not limit the study to a specific disability, health, or welfare challenge due to the potentially diverse health and wellbeing challenges facing migrant parents and their children. This opened the research up to the wide range of health and welfare challenges that exist within migrant communities.

**Table I.** Summary of the inclusion and exclusion criteria.

Included studies	Excluded studies
- Studies focusing on migrant parents’ experiences of being carers of children with health challenges and special welfare needs - Studies focusing on parents’ experiences with health and welfare delivery services - Studies focusing on migrant parents in a Nordic host country - Studies using qualitative or mixed methods that include a qualitative research methodology - Studies published from 2008 to date - Peer-reviewed primary studies - Studies published in English	- Studies exploring service providers’ perspectives on migrant parents - Studies that generally do not include parents’ perspectives - Studies only exploring experiences of parents with a Western European origin - Studies outside the Nordic region - Studies imploring only quantitative research methodology - Studies published before 200. - Grey literature (policy, proceedings)

### Search outcome

**Figure 1.**
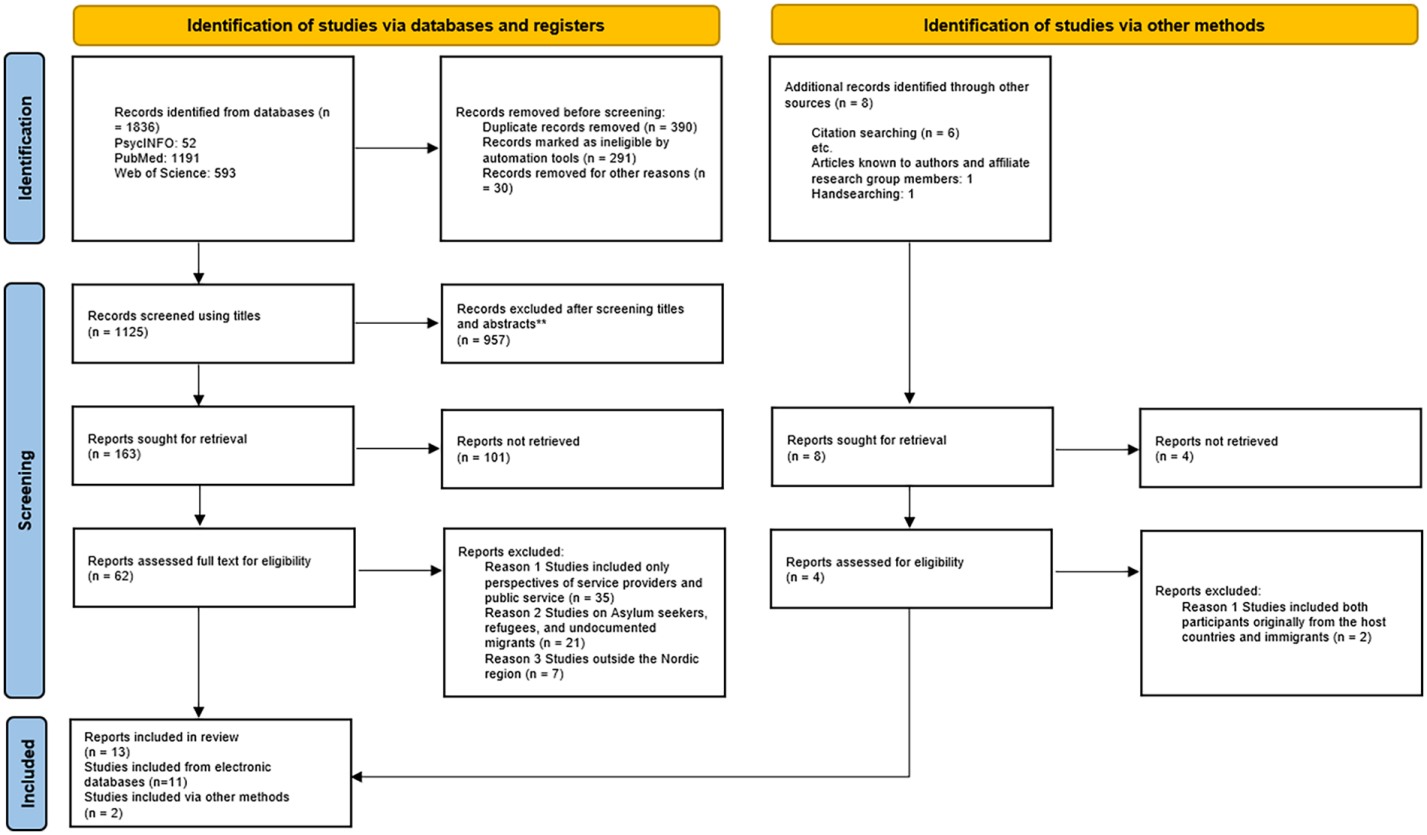
Prisma flow diagram of search output.

This summary of the scoping review focuses on the social inclusion of MCR for children with special health and welfare needs. A total of 1836 results were recorded from the three selected databases. We screened 1125 studies using titles and abstracts, and 957 were not extracted for full text assessment as they did not match the search words and inclusion criteria. Out of the 1836 study results, A total of 62 articles were read and assessed as full texts, and 13 of these were included in the final study. Of the articles included, 11 were extracted from electronic databases with the other two obtained manually through reference lists and the authors’ academic network. Figure 1 presents the search outcomes.

Studies included here were conducted in Norway [6], Sweden [3], Iceland [2], Denmark [1] and Finland [1]. These were predominantly qualitative studies [12], although one had a mixed-method design. A total of 212 MCRs were part of the included studies. Of the studies that disclosed gender, 136 parents were mothers while 67 were fathers. The majority of participants were of African, Middle Easten/Asian origins from Somalia, Liberia, Zimbabwe, Tunisia, Morroco, Iraq, Syria, Palestine and Pakistan.

### Data chart of the included studies

The included studies were ‘charted’ in order to provide a comprehensive context of the research setting [47], see attachment Table II: Characteristics of selected studies included a summary of the author’s name, year of publication, country where the study was conducted, study focus and methodology (Table II).

**Table II.** Characteristics of selected studies.

Authors and year	Country	Area/subject of focus	Sample	Methods
Arfa et al. (2020)	Norway	Experiences of immigrant parents of children with disabilities in Norway	23 parents, with six fathers and 17 mothers from non-European origins	Qualitative study, semi-structured interviews
Mangrio and Persson (2017)	Sweden	Migrants’ families experience with child healthcare system in Sweden	19 parents (14 female and five male)	Qualitative, semi-structured interviews
Arfa et al. (2022)	Norway	Participation of immigrant parents and their children in the rehabilitation programs (Welfare programs) in Norway.	23 interviews with migrant parents from non-Western countries and 17 interviews with children	A qualitative hermeneutic study, semi-structured interviews and participant observation
Kvarme et al. (2016)	Norway	Health and quality of life among immigrant parents caring for a child with complex health needs in Norway	27 parents (18 mothers and 9 fathers)	Qualitative hermeneutic study, individual and group interviews
Sajjad (2019)	Norway	Norwegian Pakistani parents’ perceptions of congenital disorders among their children	41 couples	Ethnographic study, observation, participant observation and semi-structured in-depth interviews
Heino and Lillrank (2022)	Finland	Experiences of migrant parents with a disabled child when interacting with professionals—exclusion and inclusion in Finland	20 parents from 6 different countries	Qualitative interviews
Egilson (2011)	Iceland	Parents’ perspectives on therapy services for their children living with physical disability	17 parents, 14 mothers and 3 fathers	Open qualitative interviews
Egilson et al. (2020)	Iceland	The intersection of childhood disability and migration in family lives encounter with service system in Iceland	3 mothers	Qualitative interviews
Rydström and Dalheim Englund (2015)	Sweden	Immigrant parents of children with asthma narrate meeting the healthcare system in Sweden	12 parents, 8 mothers and 4 fathers	Qualitative interviews
Povlsen and Ringsberg (2009)	Denmark	Learning to live with a child with diabetes (experiences)—problems related to immigration and cross-cultural diabetes care	6 parents in Denmark	Qualitative semi-structured interviews
Kvarme et al. (2017)	Norway	Copying strategies of immigrant parents of children with complex health needs	27 parents: 18 mothers and 9 fathers	Qualitative, focus group and individual interviews
Söderström (2014)	Norway	Lost in translation? Communication challenges in minority families’ and healthcare workers’ interactions	8 minority parents (4 fathers and 4 mothers) and 6 healthcare workers	Qualitative, in-depth interviews
Zakirova-Engstrand et al. (2020)	Sweden	Culturally diverse families of young children with ASD in Sweden: parental explanatory models	17 parents from diverse cultural backgrounds, 13 non-Western immigrant, 4 Western Swedish	Qualitative, semi-structured interviews

ASD: autism spectrum disorder.

### Limitations

One of the general methodological limitations of using a scoping review design is that it does not focus on the quality of the selected studies beyond the inclusion criteria [47]. Our study has a very broad range of (national) contexts and (disability) conditions, which may have specificities that cannot easily be compared. However, accommodating all relevant studies helps to widely mirror what is already available and, most importantly, inform the direction of future in-depth studies. Another methodological limitation is that several studies in languages other that English were excluded, which could have limited the opportunity for a wider view of the topic and area of study. Additionally, the exclusion of quantitative studies may have led to other findings being omitted.

### Synthesis of results

We use thematic analysis to present the results from the selected studies [50]. All the reviewed studies explore the experiences and perceptions of parents who have children in need of special health and welfare services in their host country. The first theme is *Initial response to having a child with special health and welfare needs.* This theme describes experiences related to how MCR responded when they gave birth to a child with special health and welfare needs. The second theme is *Encountering the Nordic health and welfare services*, which describes the MCR’s experiences of interacting with and seeking help from the health and welfare services. Finally, our last theme is *Implications of experiences for social inclusion.* In this theme, we report the impact that experiences with welfare services and caring responsibility have on the social inclusion of MCR. Table III shows the created subthemes.

**Table III.** Thematic analysis report.

Initial response to having a child with special health and welfare needs	Encountering the Nordic health and welfare services	Implications of experiences for social inclusion
- Knowledge and understanding health conditions - Social, cultural and religious response to health conditions	- Gratitude for the help and welfare services rendered - Participation versus gatekeeping and power - Cultural sensitivity of care and communication	- Feelings of shame and mental stress - Burdening family roles and responsibilities - Shrinking social network and economic opportunities

## Initial response to having a child with special health and welfare needs

Early encounters with the special health and welfare condition of children are recurrent in the reviewed literature. First, there is an indication that MCR are faced with the early responsibility of understanding what their children are going through [14, 53, 54]. Second, the studies elevate how different social and cultural values and relational backgrounds influence their experiences [7, 14, 33, 52–54]. Third, for some, seeking help and utilising services and platforms for participation was an initial response to the caring responsibility [5, 7, 18, 33, 51].

### Knowledge and understanding of health conditions

Several studies reported that migrants with caring responsibilities had early struggles in defining and understanding disability [14, 53, 54]. In a study conducted by Sajjad on genetic counselling services for Pakistani migrant parents in Norway [53], the results showed that parents’ prior knowledge and understanding of the health condition of their children contributed significantly to both their acceptance and health-seeking behaviours. In another study from Sweden, MCR admitted never having heard of autism before they moved to Sweden and, partly due to that, family members, especially fathers, were reluctant to accept their child’s condition [54]. Similar findings were recorded in Denmark where limited prior knowledge of diabetes made it difficult for MCR to come to terms with their child’s diagnosis [14]. As a result, some MCR delayed seeking help as their responses were, in some instances, tied to their knowledge and understanding of the severity of their children’s conditions [53, 54].

### Social, cultural and religious response to health conditions

The studies show that the religious beliefs, sociocultural backgrounds and family values of MCR influenced their understanding and interpretation of their children’s health conditions [7, 14, 52–54]. Cultural and religious beliefs and family values were reported as contributing factors to the complexity of MCR perceptions of their children’s conditions [5, 14, 53, 54]. These perceptions involved questions and conclusions concerning why they ended up with a disabled child, with others insinuating the presence of a curse, God’s will or an act of evil [53, 54].

## Encountering Nordic health and welfare services

The included studies generally showed the experiences of MCRs at the individual and personal level, and also their interaction with health and welfare services. Some parents showed concern for their children’s developmental delays and sought help earlier, as found in studies from Finland [6], Norway and Sweden [33, 54]. The caring responsibility was also a major reason for migrating away from or staying in host countries in order to seek help in what they regarded as a better functioning health and welfare system [6, 28]. Parents showed motivation to provide care for their child by seeking information and utilising services and platforms for participation [14, 33, 51]. These interactions culminated in different perceptions and attitudes on their overall experiences with the services offered.

### Gratitude for the help and welfare services rendered

In seven of the studies, MCR expressed gratitude for the health and welfare services rendered to them [4, 5, 7, 12, 14, 28, 51]. This appreciation stemmed from various experiences, among them viewing their migrant status and the benefits that came with it as some form of favour from their host countries [4, 7, 12] and comparing services rendered in the host country with those of their home countries [7, 12]. For others, a good relationship with health and welfare professionals and opportunities for participation created a sense of being respected and cared for [4, 5, 14, 51]. In some instances, MCR were pleased by the accessibility and information dissemination strategies such as the use of pictorials as reported in Iceland [14, 28]. However, Rydström and Dalheim Englund reported that, due partly to this sense of gratitude, MCR in Sweden became reluctant to seek additional financial help or were hesitant to critique or complain about their relationship with healthcare providers [4].

### Participation versus gatekeeping

Although at face value the migrant parents were appreciative of the health and welfare services, they also lamented the professional and structural barriers to their participation in them [5–7, 12, 14, 28]. In a study on MCR in Finland by Heino and Lillrank [6], MCR reported how professionals politely listened to their opinions, but then ignored them when making treatment decisions and, in the worst cases, did not invite them to any treatment discussion. In an extreme case in Iceland, a migrant parent felt that staying in Iceland was no longer an option due to the discrimination she believed she had been subjected to [28]. The studies generally stimulate debate on the implications of the nonparticipation of MCR and whether health professionals should strictly follow medical and clinical dictates over the inclusion of parents’ non-clinical opinions on their children’s welfare [7, 26, 28].

### Cultural sensitivity of care and communication

The availability of culturally sensitive information and communication channels, and lack thereof, resulted in different experiences and outcomes for MCR [7, 51–53]. Most of the parents involved in the studies faced linguistic difficulties, limiting their chance to interact with and utilise the welfare services [7, 14, 52, 55]. For instance, in Norway, MCR were unable to express themselves in a way that would be likely to make Norwegian health professionals take them seriously because the expression of illness or disease reflected their own culture and was less influenced by Norwegian nor western medical thinking [51]. Heino and Lillrank also found that these information and communication challenges further minimised opportunities for the social inclusion and recognition of MCR in Finland [6].

However, what also emerged in the findings is that the parents’ experiences are further dependent on the professional attitude of health workers handling their cases [4, 28]. In Sweden, Rydström and Dalheim Englund found that, even though other MCR raised concern over the competence of professionals in understanding their unique situations, other parents spoke highly of the health and welfare providers and built healthy professional relationships with them [4]. Several studies recommended that health and welfare professionals facilitate knowledge about how to obtain help, who could help, and what their rights were in caring for their child [4, 7, 14, 18].

## Implications of experiences for social inclusion

The experiences of having a child with special health and welfare needs brought significant changes to MCR. These changes resulted in feelings of shame and mental stress, changing family roles and responsibilities, a shrinking social network and economic opportunities.

### Feelings of shame and mental stress

In several studies, it was noted that a health diagnosis of a condition in migrant children resulted in negative implications for the wellbeing of most MCR [5, 14, 18, 33, 53]. Encountering shame and mental stress are reported both during the initial responses to the health and welfare conditions and when meeting with health and welfare services [33, 53]. The constant distress is highlighted as negating the individual help-seeking behaviour of the MCR, resulting in self neglect, poor quality of life and exclusion [18, 33].

### Burdening family roles and responsibilities

The reviewed studies generally acknowledged the impact of the children’s health and welfare condition on family life and relationships [7, 14, 18, 28, 33, 53]. For instance, changes in parental roles significantly affected spousal relations and responsibilities, as reported in Norway and Denmark [14, 18]. Studies from Iceland, Norway and Sweden also found that the responsibility of care became more gendered [7, 18, 28], with mostly mothers taking the primary caregiver role while fathers started working more hours and spending less time with the family. The gendered response to care resulted in some mothers facing fragile economic situations [6, 7, 14, 18].

### Shrinking social network

Caring for a child with a special health condition is further reported to have caused the shrinking social network for parents with caring responsibilities [5, 7, 28]. Opportunities to build social relationships became minimal for most parents and almost non-existent for mothers who, in most instances, took up the primary caregiver role, as reported in Iceland and Norway [18, 28, 53]. The challenging networking experiences and level of inclusion in social settings further affected migrants ability to learn more about the host country’s culture, including local languages [28].

## Discussion

The aim of this review was to explore and summarise the emerging themes in existing peer-reviewed studies on the personal experiences of migrant parents of children with special health and welfare needs and their interaction with health and welfare services in the Nordic countries. Our findings contribute to the broader understanding of the varying experiences of MCR in the context of previous peer-reviewed studies and how that may explain the opportunities and extent of their inclusion/exclusion. This section will now discuss the most important aspects from our findings. These discussion themes are *Access to public health and welfare services* and *Exploration of opportunities for social inclusion.* These themes are interpreted and discussed within the dictates of social inclusion because of their direct link to participation, access and empowerment in the utilisation of health and welfare services for migrants.

### Access to public health and welfare services

The results show a general sense of gratitude by MCR for the health and welfare services received compared with what they receive in their home countries [5, 7, 28]. Migrants’ positive remarks regarding the health and welfare services can be attributed mostly to the structural efficiency, quality and accessibility of the Nordic welfare systems compared with other countries [5, 7, 24, 28].

The migrants’ gratitude for the services could also have emanated from the socio-political sentiments of their identity as migrants and overall acceptance in society. For some, receiving services would appear as a privilege because of the point of deficit or lack of access to basic amenities in their home countries [38, 43]. This can be explained by the fact that most MCR seldom viewed access as a basic human right while others were not asking for more than what they were offered. Such attitudes to care services could be linked to the inequalities that may arise between migrants compared with the native majority population on the basis of their help-seeking behaviour [5, 17, 56].

The included studies show that access to health and welfare services and general experiences of MCR were affected by the availability and/or lack of relevant information and communication about their roles and rights to services. Cases of limited information about diagnosis and MCR’s understanding of the reasons or role in their involvement in health and welfare is also reported in Canada and the Netherlands [17, 57]. Lim et al. links such information gaps to a lack of health literacy [11], arguing that this hinders MCR’s ability to obtain and use information on available services. Similar conclusions were made in Netherlands, where MCR’s familiarity with terms used in identifying disability and knowledge of what services are available proved salient [17]. Strengthening formal and informal communication between MCR and welfare professionals could provide opportunities for quality and relevant services as echoed by Nyikach et al. [58].

Our findings can further be explained within the theory of social inclusion, which links access to interventions and resources that target groups described as disadvantaged and with particular needs, including access to relevant and appropriate information on the available resources [36, 43]. This is from the background that limited access to information presents a limitation that needs redress. This is because access to information is a contributing factor to the capabilities required to capitalise on opportunities for social inclusion [41]. In this pretext, our findings demonstrate that social inclusion is guaranteed not only by the availability and rights to services but also by the knowledge of their availability, supportive social and professional relationships and families [38, 43]. We argue that such identity backgrounds of migrants have implications for their access to and maximisation of health and welfare services.

Scholars in social work and health service development have recommended that health professionals have relevant knowledge and cultural know-how to understand service users and find appropriate ways to communicate and provide suitable services [42, 59, 60]. Hutchinson and Lee describe this as ‘equality in diversity’ and a way of levelling access to welfare resources and inclusion for MCR [42]. This can include collecting and evaluating information within a context involving MCR in order to identify barriers and improvement in policy and practice [35]. In that sense, welfare professionals have the responsibility to open opportunities for individuals’ understanding of health information regardless of their background [61].

Several proponents of empowerment as a key strategy in welfare inclusion suggest that the users of services should have the right to not only participate but also to gain knowledge and the capacity to influence how services are delivered to them [37, 40, 62]. Eide et al. conceptualise that this empowering process has to be continuous, participatory and interactive [39]. In that sense, continuous interaction between MCR and health and welfare services’ providers have the potential to yield positive outcomes towards social inclusion.

### Exploration of opportunities for social inclusion

The synthesised results show MCR’s greater desire for participation in decision making, and being recognised as equal partners by both the welfare system and individual health and welfare professionals [6, 7, 51, 52]. However, our findings demonstrate how MCR can feel disempowered and unrecognised in instances where the welfare systems are structured and expert driven [6,28]. This could result in limited opportunities for participation and social inclusion as similarly found in the UK, Canada and the Netherlands [17, 34, 63]. In their qualitative synthesis of migrant parents experiences of accessing health and welfare services, Karim et al. found that some migrant parents felt patronised and disrespected [64]; thus, their attitudes to services in host countries negatively changed. What these varied experiences by MCR might entail is that there may be a need to open opportunities for participation through balancing between health and welfare providers’ regulatory and facilitatory roles in providing services.

Our observations from the included studies further points towards a general link between experiences of MCR with the welfare system and their trust levels [12, 14, 65]. There are chances that MCR may choose to migrate back to their home countries or seek alternative care elsewhere in situations where there are tensions or misunderstandings with the health and welfare services [28]. Such circumstantial migration back to one’s home country could be a demonstration of a lack of trust between the welfare services and MCR. In contrast, other studies have generally attributed MCR’s longer stays in host countries as a coping mechanism to access better health alternatives and conditions compared with their home country [63, 66]. These different experiences demonstrate the intersection between social inclusion, migration and being a MCR [28].

Our results synthesis also elevates the significance of family life and relationships regarding how MCR respond to, and cope with, their caring responsibility. Corresponding to other studies in Canada and Greece [57, 67, 68], our results show that, in many instances, social networks shrink, and family relations and roles change between genders [12, 28, 33] because of the caring responsibility. Scholars argue that this change in family roles and networking opportunities contributes to differences in outcomes for migrant men and women with caring responsibilities [28, 67]. What could explain this divide are social values, religious beliefs and cultural expectations over the role of women in providing care and men providing families’ financial security [53, 68, 69]. We argue that participation and social exclusion is multidimensional and therefore linked to multiple social determinants including gender, economic situation and migration status [60]. From this background, an intersectional approach to social inclusion of MCR could be viable in both research and practice.

### Implications for public health and welfare practice and research

The findings generally show the challenges that MCR face balancing between their caring responsibility and meeting the health and welfare needs of themselves and their children. Opportunities for participation for MCR, both as carers and individual members of society, are vital to social inclusion. This is because children are likely to be impacted if parents face challenges integrating into society [70, 71]. As such, an attempt to explore family life and relationships, opportunities for participation and knowledge about the welfare system could be of primary interest in fostering the social inclusion of MCR. This is because the outcome and experiences of MCR were found to be linked not only to their interaction with the welfare services but also how they harness the social resources around them such as family, social networks, and information. Our observations may thus contribute to health promotion, particularly regarding how individual and family circumstances such as migration status, available community resources and welfare systems affect the wellbeing and inclusion of vulnerable groups in societies. All these are key social determinants of health and wellbeing that may require intersectoral collaboration. An exploration of these social determinants may thus have the potential to answer some public health and welfare inequality challenges in the Nordic region.

## Conclusion

In conclusion, the experiences of MCR are linked to their initial response to having a child with special health and welfare needs, encountering the health and welfare system and overall inclusion in their host countries. Findings showed that MCR acknowledge the opportunities presented by the health and welfare services in the Nordic regions as evidenced by their sense of gratitude. The Nordic countries have accessible, affordable and well-equipped infrastructure for health and welfare services compared with the home countries of most MCR. The reviewed studies show that different challenges to participation faced by MCR threaten their wellbeing, as well as influence their coping strategies and opportunities for social inclusion. Further exploration of strategies for the social inclusion of MCR could be relevant to informing future research and public health and welfare services development.
